# Access to cervical cancer care in sub-Saharan Africa: a narrative review of barriers and opportunities

**DOI:** 10.3389/fpubh.2026.1881652

**Published:** 2026-08-18

**Authors:** Netsanet Mengistie, Himanshu Verma, Alessandro Bozzon, Wichor M. Bramer, Vera Dubbink, Marlieke de Fouw, Jan Carel Diehl

**Affiliations:** 1 Faculty of Industrial Design Engineering, Delft University of Technology, Delft, Netherlands; 2 Knowledge and Intelligence Design, Delft University of Technology, Delft, Netherlands; 3 Medical Library, Erasmus University Medical Center, Rotterdam, Netherlands; 4 Department of Gynecology, Leiden University Medical Center, Leiden, Netherlands

**Keywords:** access to care, barriers, cervical cancer care, opportunities, sub-Saharan Africa

## Abstract

**Background:**

This narrative review examines the barriers and opportunities that shape access and delivery of cervical cancer care services in sub-Saharan Africa. Unlike prior studies that only focused on single phases of care, this review adopts a comprehensive perspective by assessing access across the full cervical cancer care continuum.

**Methods:**

A narrative review was conducted using six databases (Embase, Medline, Web of Science, Scopus, African Index Medicus, and CINAHL Plus). The included studies were thematically analyzed, and emerging themes were interpreted through the lens of the five dimensions of healthcare access: accessibility, availability, affordability, acceptability, and accommodation.

**Results:**

Across 38 included studies, findings identified three overarching categories of barriers. (1) Individual-level barriers, including knowledge and awareness, socio-demographic and economic factors, and attitudes and beliefs; (2) Community-level barriers, including cultural acceptance and social norms, and community and social support networks, which further constrained engagement with services; and (3) Health-system-level barriers, including resource limitations, service delivery and quality, and weak policy implementation, which compounded delays and limited care effectiveness. Moreover, opportunities, such as methodological opportunities (community-driven health education, culturally informed healthcare interventions, and multi-stakeholder engagement) and technological opportunities (technology-enhanced health education, digital systems for patient navigation, and task decentralization using mobile health platforms, telehealth, electronic health records, and digital reminder systems), could be effectively integrated into the healthcare system to improve access and service delivery.

**Conclusion:**

Access to cervical cancer care in sub-Saharan Africa is shaped by interconnected structural, sociocultural, and individual barriers that extend beyond supply and service availability alone. Literature often focuses on screening, diagnosis, and treatment of cervical cancer, while research gaps identified in this review persist in primary prevention, follow-up, and palliative care services. Findings from the review suggest that integrated, context-specific, and human-centered healthcare interventions are needed to strengthen care coordination, improve access, and enhance care outcomes.

## 1 Introduction

**Figure 1:**
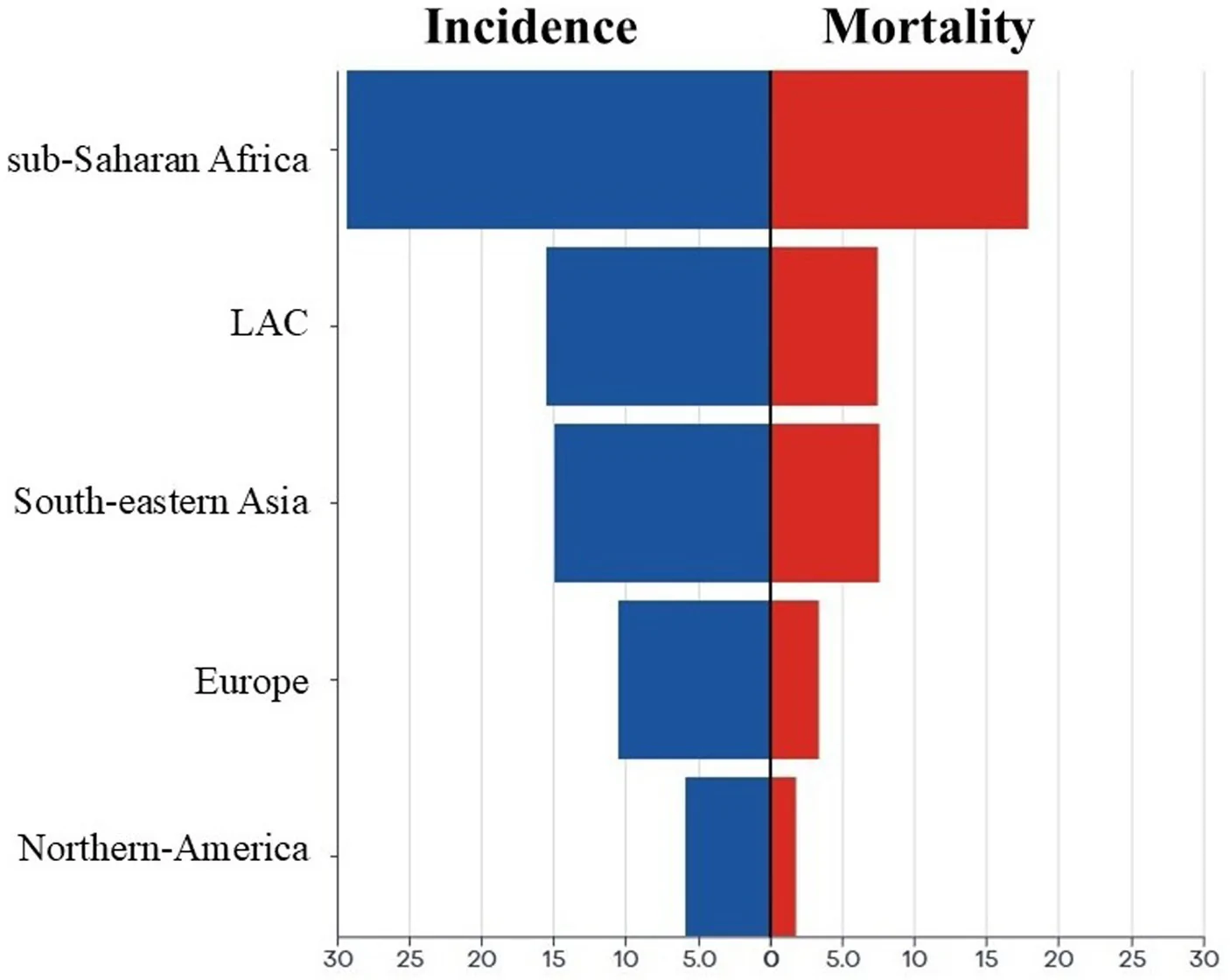
Age-standardized rate (World) per 100,000, incidence and mortality of cervical cancer, in 2024 (8).

Cervical cancer, primarily caused by persistent infection with a high-risk type of human papillomavirus (HPV) (1), is the most common cancer among women worldwide, with an estimated 604,000 new cases and approximately 280,000 deaths in 2024 (2). It remains a major public health concern, particularly in low-resource settings (LRS), where nearly 84% of new cases and 88–90% of cervical cancer-related deaths occurred in these settings (3–5), where sub-Saharan Africa (SSA) bears the highest burden, followed by Latin America and the Caribbean (LAC) and Southeast Asia (6, 7) as shown in Figure 1 (8). This distribution underscores substantial inequities in access to prevention, early detection, and treatment (9–11), reflecting persistent gaps in health system capacity and service delivery across the cervical cancer care continuum.

Access to cervical cancer services in SSA remains limited and inconsistent, reflecting broader health system barriers in LRS (12, 13). Access extends beyond mere service availability; even where services exist, they are often fragmented, poorly coordinated, and unevenly distributed (14). Consequently, many women experience delayed diagnosis, limited treatment options, and suboptimal follow-up care, resulting in low uptake of guideline-adherent and cancer-directed therapy (15, 16). These barriers are further exacerbated for women in rural and remote areas, as well as for refugees and displaced populations, who face additional geographic, financial, and structural hurdles to care (17, 18). Such inequities highlight both systemic inadequacies and within-system disparities, underscoring the need to strengthen and integrate cervical cancer care across all levels of the health system (17, 19, 20).

Existing literature identifies multiple, interrelated barriers that contribute to gaps in cervical cancer care in SSA. These include limited health literacy and awareness (21–23), inadequate access to healthcare facilities (23–25), and insufficient service and infrastructure for prevention, diagnosis, and treatment, such as HPV testing, cytology services, pathology and imaging capacity, and radiotherapy equipment (26–29). Financial constraints also impede the scale-up of HPV vaccination, screening programs, and diagnostic services, while shortages of trained and specialized healthcare workers further restrict service delivery (30). Collectively, these constraints contribute to fragmented care pathways, low screening coverage, delayed diagnosis, and poor treatment outcomes across the region (29, 31).

Beyond structural and health system-level limitations, gender inequality and sociocultural factors further shape women’s access to cervical cancer care services in SSA. In male-dominated healthcare systems, women may feel discomfort or lack agency when seeking care from male providers (32). Social norms that restrict women’s decision-making power, limit their control over financial resources, assign responsibilities for household duties, and prioritize male authority often delay or entirely prevent them from seeking care (33). Context-specific evidence from countries such as Ethiopia and Ghana illustrates how cultural expectations, restricted mobility, provider bias, and discriminatory practices, including obstetric violence, can further constrain timely access to essential healthcare services (34, 35).

Previous research has largely focused on individual phases of the cervical cancer care continuum, such as either screening, diagnosis, treatment, or follow-up, often examining barriers in isolation. While these studies provide valuable insights, they offer a limited understanding of how barriers arise, interact, and evolve throughout the care pathway, or how they can be addressed in a coordinated manner. As a result, existing evidence remains fragmented, with insufficient attention to how barriers operate across different levels of the care system. More integrative approaches are therefore needed to better understand these dynamics and to map the cervical cancer care ecosystem, including the relationships between barriers and potential points for intervention across the continuum of care.

Therefore, this narrative review aims to synthesize existing evidence on barriers to access and delivery of cervical cancer care services across the continuum of care in SSA. In addition, it examines opportunities suggested and recommended in the literature for addressing these barriers, with particular attention to methodological approaches and the role of digital health technologies. The review addresses the following research question: *What does the existing literature reveal about access to and delivery of cervical cancer care services in sub-Saharan Africa, and which contextual barriers and opportunities shape access to these services?*

## 2 Methods

### 2.1 Study design

A narrative review employing a systematic literature search approach was conducted to identify barriers to accessing and delivering cervical cancer care services, as well as opportunities to improve service delivery in SSA. A narrative review was considered the most appropriate design because the primary objective was to synthesize and interpret existing evidence across the cervical cancer care continuum, rather than to comprehensively map the breadth and characteristics of the available evidence. To enhance methodological rigor, transparency, and reproducibility, a systematic literature search, predefined eligibility criteria, and Preferred Reporting Items for Systematic Reviews and Meta-Analyses (PRISMA)-guided procedures were used to report the study identification and selection process. The evidence from the included studies was then synthesized narratively to provide an integrated understanding of the complex factors influencing access to cervical cancer care in SSA, including accessibility, availability, acceptability, financial affordability, and accommodation.

To analyze findings across selected studies and to understand the phenomena of healthcare access, the thematic synthesis was guided by a pragmatic approach (36) and a critical realist philosophical stance (37). This position recognizes that barriers to healthcare access are both empirical realities and socially constructed phenomena. Together, these perspectives informed the formulation of the research question and guided the interpretation of findings. The Theory of Access (38) provides a pragmatic lens for analyzing the functional fit between health service users and the health system, emphasizing practical dimensions of accessibility (geographic location of providers or consumers and ease of reaching them), availability (presence of necessary resources, providers, and facilities), affordability (capacity of consumers to pay for services without financial hardship), acceptability (compatibility between consumers expectations and the characteristics of providers), and accommodation (resources organized to needs). In contrast, the Social Determinants of Health Framework (SDH) (39) offers a practical understanding of how socio-structural factors, such as gender norms, educational attainment, and socio-economic inequalities, shape this dimension of healthcare access. Together, these two frameworks provide a comprehensive view of access, extending beyond supply-related issues to encompass the contextual barriers that drive differences and inequalities in healthcare, thereby offering a holistic understanding of the barriers and opportunities for improvement.

Other frameworks, such as the Health Belief Model (40), Andersen’s Behavioral Model (41), and the Socio-Ecological Model (42), were not included because they tend to emphasize narrower or single-level perspectives; e.g., a focus on why individuals do or do not engage in preventive behavior, primarily at the personal and household levels, and a lack of operationalization of how determinants interact to shape access. These perspectives may not fully capture the systemic and contextual barriers that shape access and service delivery in SSA.

### 2.2 Search strategy

The literature search was conducted across six databases: Embase via embase.com, Medline via Ovid, CINAHL Plus via EBSCOhost, Web of Science, Scopus, and African Index Medicus to identify relevant resources. The search used the main terms “cervical cancer,” “accessibility,” and “sub-Saharan Africa,” combined with related keywords such as delivery, disparity, barrier, problem, challenge, equality, inequality, facilitator, opportunity, and names of specific SSA countries, as shown in Table 1. The search strategy was developed in May 2025 by W. M. B., a biomedical information specialist experienced in systematic reviews, and N. M.

**Table 1:** Constructed search terms used in the database searches.

Key concepts	A combination of search terms used across databases
Cervical cancer	(uterine cervical neoplasms / OR (((cervix* OR cervical*) ADJ6 (cancer* OR tumor* OR tumour* OR malignan* OR carcino* OR adenocarcino* OR adenoma* OR neoplas* OR lymphoma* OR leiomyosarcoma* OR sarcoma* OR oncolog*))).ab,ti,kw. OR (((cervix* OR cervical*) AND (cancer* OR tumor* OR tumour* OR malignan* OR carcino* OR neoplas* OR lymphoma* OR leiomyosarcoma* OR sarcoma* OR oncolog*))).ti.)
Access/Accessibility *(including barriers and opportunities)*	(health services accessibility / OR healthcare disparities / OR * delivery of health care / OR (((care OR healthcare OR treatment* OR therap* OR medication* OR service* OR screening* OR diagnos*) ADJ6 (access* OR affordab* OR availab* OR disparit* OR inequalit* OR equalit* OR inequit* OR equit*)) OR ((care OR healthcare OR treatment* OR prevent* OR therap* OR medication* OR service* OR screening*) ADJ6 (deliver*) ADJ6 (challenge* OR problem* OR barrier* OR facilitator* OR opportunit*))).ab,ti,kw. OR (accessib* OR affordab* OR availab* OR challenge* OR ((care OR healthcare OR treatment* OR prevent* OR therap* OR medication* OR service* OR screening* OR diagnos*) ADJ6 (deliver* OR barrier* OR facilitator* OR opportunit*)
sub-Saharan Africa	(Africa South of the Sahara / OR * Africa / OR ((Africa ADJ3 south ADJ3 Sahara) OR ((sub-sahara* OR subsahara*) ADJ3 africa*) OR Angol* OR Benin* OR Botswan* OR Burkina-Faso* OR Burundi* OR Cameroon* OR Cape-Verd* OR Central-Africa* OR Chad* OR Comor* OR Congo* OR Cote-d-Ivoire* OR Ivory-coast* OR Djibout* OR Eritrea* OR Eswatini* OR Ethiopia* OR Gabon* OR Gambia* OR Ghana* OR Ghanese* OR Guinea* OR Kenya* OR Lesotho* OR Liberia* OR Madagascar* OR Malawi* OR Mali OR Malinese OR Mayotte* OR Mozambique* OR Namibia* OR Niger* OR Rwand* OR Sahel* OR Senegal* OR Sierra-Leone* OR Somali* OR (South* ADJ3 Africa*) OR Sudan* OR Tanzania* OR Togo* OR Uganda* OR Zambia* OR Zimbabw* OR zaire*)

### 2.3 Eligibility criteria

The inclusion and exclusion criteria were guided by the objective of identifying evidence on the ease or difficulty of obtaining cervical cancer care services in SSA. In this review, access was conceptualized as a multidimensional construct encompassing the 5A’s of physical availability and service presence, geographical accessibility, financial affordability, acceptability, and accommodation, consistent with the Theory of Access and frameworks addressing the Social Determinants of Health (38, 39). Eligible studies included peer-reviewed qualitative, quantitative, and mixed-methods research; conference proceedings with full texts; and relevant book chapters that addressed access to cervical cancer services across one or more phases of the care continuum. Studies published in English between 2015 and 2025 were included to capture evidence aligned with recent cervical cancer prevention and control strategies. Conference abstracts without full texts, editorials, commentaries, literature focusing exclusively on biological or clinical aspects of cervical cancer, non-English publications, and literature conducted outside the SSA context were excluded.

### 2.4 Study selection

**Figure 2:**
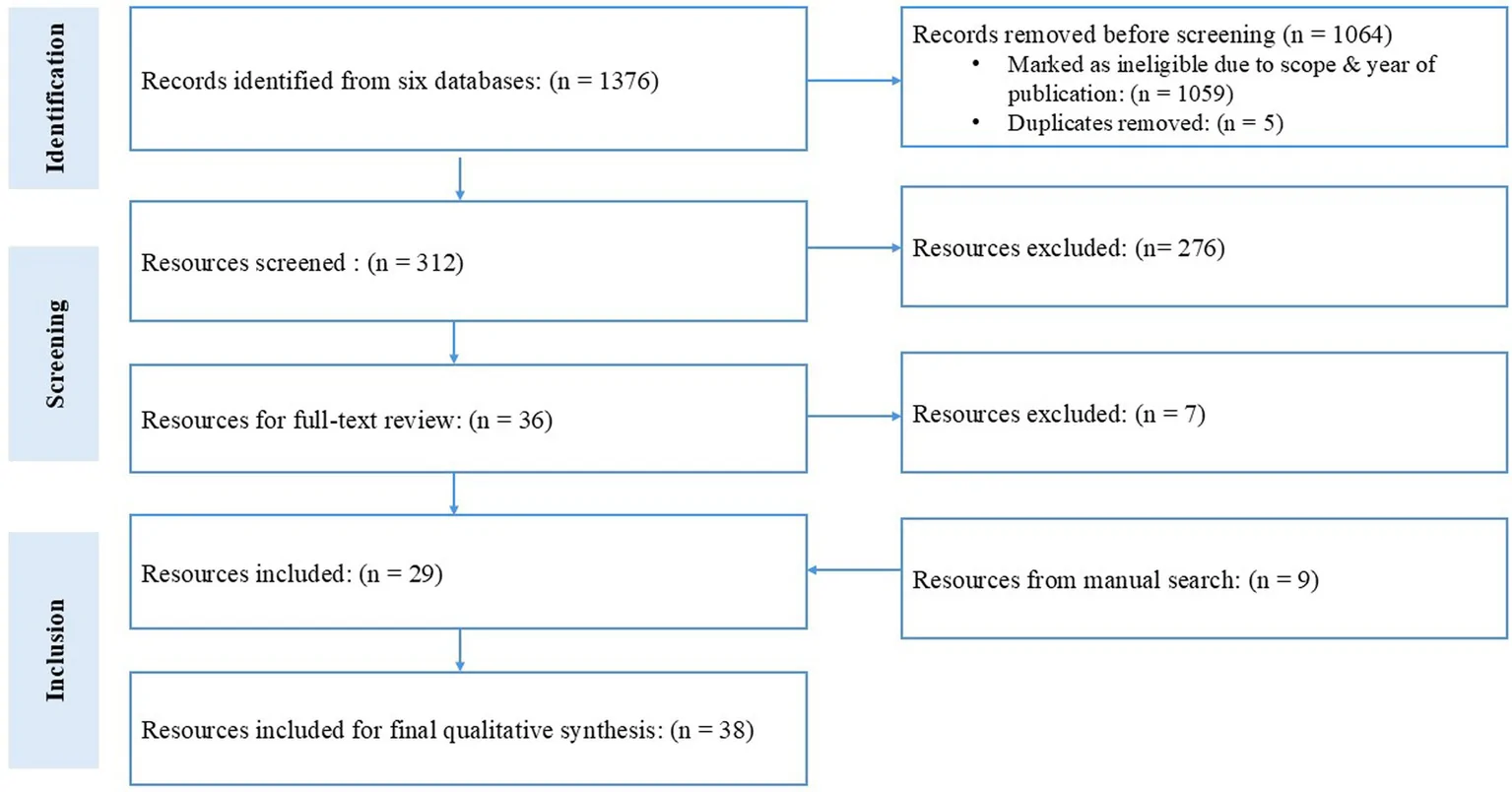
PRISMA flow diagram of included studies in the review results.

The narrative review was conducted in accordance with the PRISMA guidelines, as outlined by Moher et al. (43), and inclusion and exclusion decisions were made using Covidence.^1^ A total of 1,376 resources were initially identified from the electronic databases, of which 1,064 were ineligible for screening due to duplication, publication year, study population, or being out of scope for the review. Consequently, 312 studies were screened; 36 were eligible for full-text review, and the remainder were excluded because their topics were not relevant to the research question. Finally, 29 studies met the inclusion criteria; seven studies were excluded because they focused primarily on clinical and biomedical aspects, such as vaccine dosage, screening protocols, and medication quality, rather than on the 5A’s of accessibility. An additional nine relevant resources were identified through manual searches conducted during data extraction. In total, 38 studies were included in the final qualitative synthesis (see Figure 2).

## 3 Results

### 3.1 Characteristics of included studies

**Figure 3:**
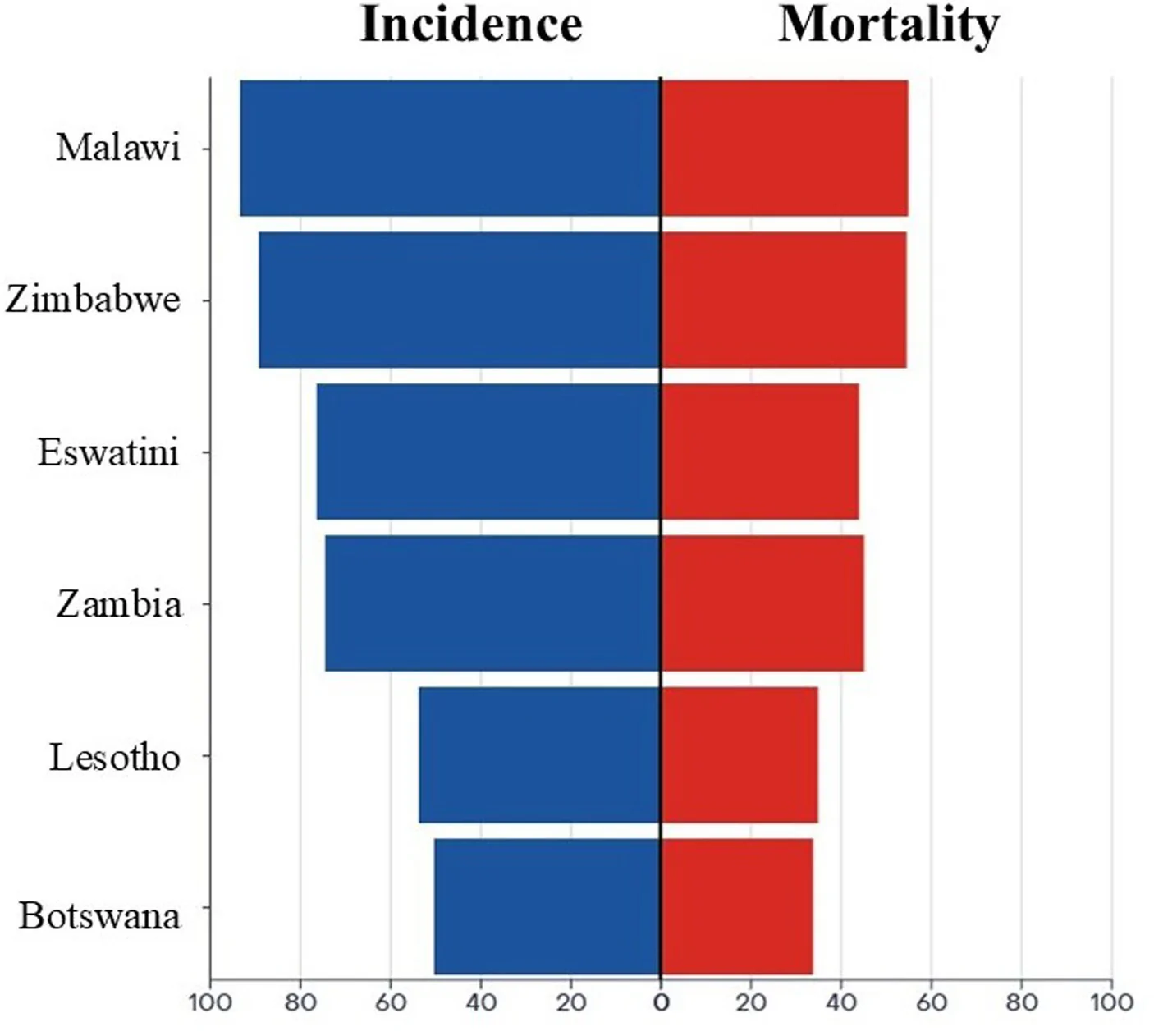
Age-standardized rate (SSA) per 100,000, incidence and mortality of cervical cancer, in 2024 (8).

There was significant variation in the types of articles that were included in this review. Among the 38 included studies, 44.7% (*n* = 17) employed a quantitative design using surveys, controlled quasi-experimental methods, and retrospective methods; 34.2% (*n* = 13) were qualitative studies based on interviews, a focus group discussion, and reviews and; 21% (*n* = 8) used mixed methods, including semi-structured interviews, surveys, desk reviews, and a focus group discussion. The majority (52.63%) of the included studies were conducted in Eastern Africa (Zimbabwe = 5, Kenya = 5, Ethiopia = 4, Tanzania = 2, and Malawi, Mozambique, Rwanda, and Uganda = 1 each). In fact, although Malawi, Eswatini, and Zambia have the highest incidence and mortality rates of cervical cancer (see Figure 3; (8), relatively few studies have been conducted in these countries, possibly reflecting limited research capacity and health system resources.

### 3.2 Geographic distribution of included studies

The geographical distribution of the included studies was classified according to the United Nations Statistics Division (UNSD) M49 geographical classification of Africa, which categorizes SSA countries into Eastern, Middle (Central), Western, and Southern Africa (44). The 38 included studies represented all four geographical regions of SSA. Seven studies adopted a multi-country perspective across SSA and were therefore considered separately as the fifth category for this study. Of the remaining 32 single-country studies, the largest proportion was conducted in Eastern Africa (*n* = 20), followed by Western Africa (*n* = 6), Southern Africa (*n* = 2), and Middle (Central) Africa (*n* = 3), as shown in Table 2.

**Table 2:** Geographic distribution of included studies across SSA.

SSA region	Countries represented	Number of studies
Eastern Africa	Kenya and Zimbabwe (*n* = 5 each), Ethiopia (*n* = 4), Tanzania (*n* = 2), Malawi, Mozambique, Rwanda, and Uganda (*n* = 1 each)	20
Southern Africa	South Africa and Botswana (*n* = 1 each)	2
West Africa	Nigeria (*n* = 4) and Ghana (*n* = 2)	6
Middle (Central) Africa	Cameroon (*n* = 3)	3
Multi-country perspective	Studies conducted across multiple countries in SSA	7
Total: 38		

### 3.3 Scope of included studies

**Figure 4:**
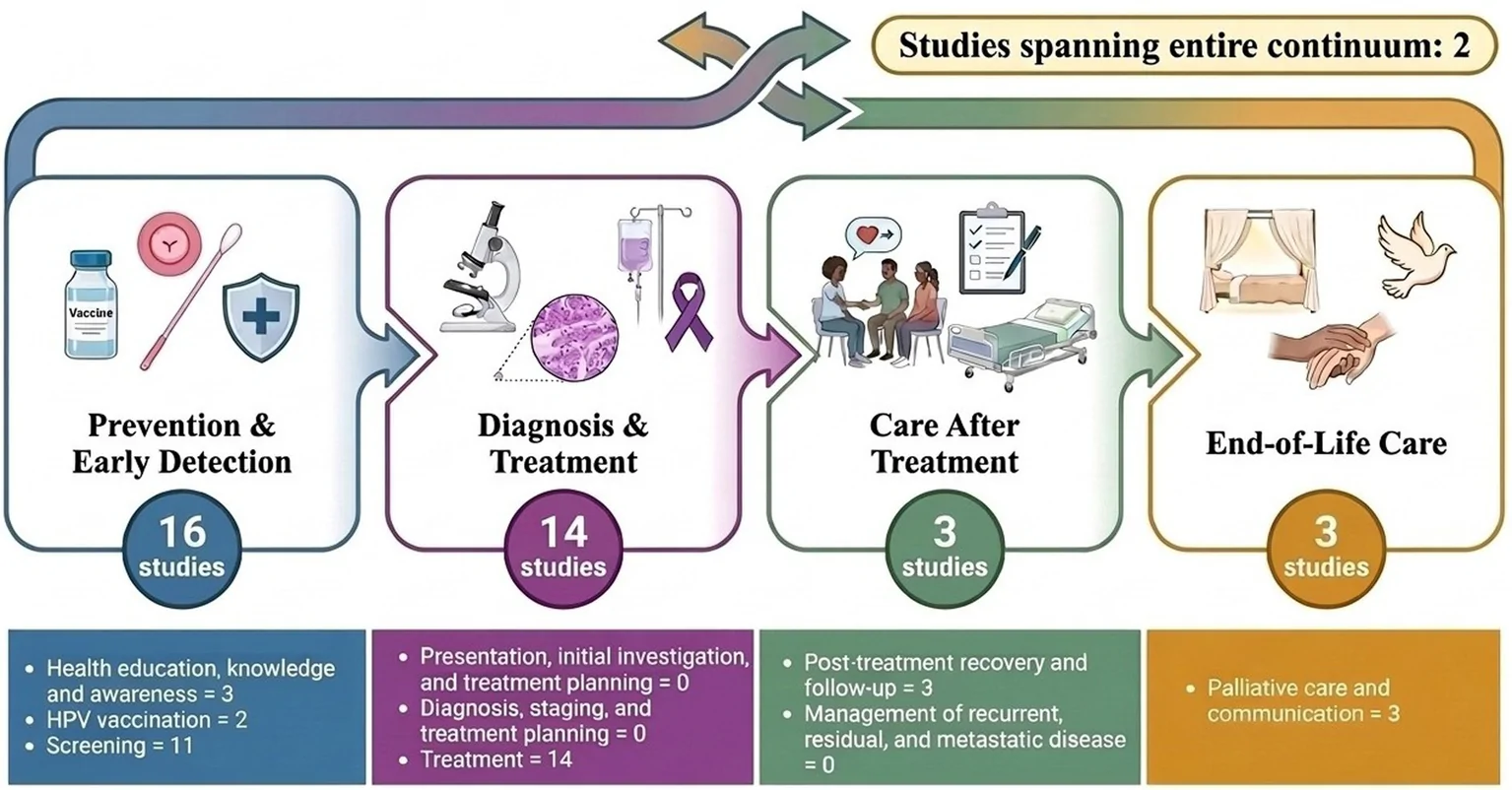
Scope and study focus of included studies in a cervical cancer care continuum.

Using a continuum-of-care framework, cervical cancer care was conceptualized into seven phases: prevention and early detection (including HPV vaccination and screening); presentation, initial investigation, and referral; diagnosis, staging, and treatment planning; treatment; post-treatment recovery and follow-up; management of recurrent, residual, and metastatic disease; and end-of-life care (including palliative care and communication) (45). To align with the scope of the included studies, these phases were grouped into four broader categories: *prevention and early detection*; *diagnosis and treatment*; *care after treatment*; *and end-of-life care*. Additionally, another phase category emerged, focusing on health education, knowledge, and awareness of cervical cancer, its symptoms, and treatment options, which are essential components of an effective cervical cancer prevention and control program (46, 47). Of the 38 included studies, three addressed health education, knowledge, and awareness; two focused on HPV vaccination; eleven on screening; fourteen on diagnosis and treatment; three on follow-up care; three on palliative care; and the remaining two focused on the entire care continuum, as shown in Figure 4. Examination of the distribution of studies across this continuum revealed an imbalance in scholarly attention. The remaining two studies examined opportunities to improve access and service delivery of cervical cancer care.

The existing literature is largely focused on screening, diagnosis, and treatment, with comparatively limited attention to health education, knowledge, and awareness, follow-up, palliative care, and communication services. This imbalance reflects a predominant focus on early detection and clinical curative management, while insufficiently addressing other critical phases of care that influence patient outcomes and continuity of care. Limited research highlights gaps in understanding preventive strategies beyond HPV vaccination and screening, as well as care after treatment and end-of-life care, which remain underexplored in this context.

### 3.4 Data extraction and theming

Data were extracted from the 38 included studies, and findings were categorized into both predetermined and emerging themes, following Braun and Clarke’s principles of thematic analysis (48). Initially, the first author reviewed all included studies to ensure familiarity with the content and developed a structured data-extraction table to guide data extraction. The fifth author independently conducted the initial data extraction. Subsequently, the first author reviewed and refined the extracted data to ensure completeness and conducted the thematic analysis. The data extraction procedure focused on two main categories of identifying barriers and opportunities related to access to and the delivery of cervical cancer care services, as presented in the Supplementary file. The first category captured documented barriers that hindered, influenced, or limited communities’ access to cervical cancer care services, as well as barriers affecting healthcare providers’ ability to deliver these services; whereas the second category focused on opportunities, defined as approaches, strategies, or tools reported in the literature that could facilitate access to care or help address identified barriers. These opportunities were most commonly identified in the discussion and recommendation sections of the included studies, where authors described them as opportunities or facilitators for improving service delivery and access.

Following data extraction, a hybrid inductive-deductive thematic approach was used to categorize findings for qualitative synthesis. Eight barrier sub-themes were identified: health education, knowledge, and awareness; socio-demographic and economic factors; attitudes and beliefs; cultural acceptance and social norms; social support networks; resource limitations; service delivery and quality; and policy and governance. These sub-themes were subsequently organized into three overarching barrier themes that represent broader layers of influence on access and delivery of cervical cancer care services, as presented in Table 3. Because barriers do not fall into a single theme, the sub-themes are not explicitly discussed in the results and discussion section. Instead, it is suggested that they be integrated into the document in a less ordered manner. This broader-level thematic clustering was undertaken to provide a transparent, evidence-informed structure for understanding how barriers operate across multiple levels of the health system and social context. Although grouped analytically for clarity, the barriers frequently intersect and interact in practice, and the reviewed studies consistently indicate that barriers to access are interdependent rather than isolated.

**Table 3:** Theme categorization for identified barriers.

Themes for barriers	Sub-themes	Studies
Individual-level barriers	Health education, knowledge, and awareness; socio-demographic and economic factors; attitudes and beliefs	Rendle et al. (49), Randall and Ghebre (50), Ngalla et al. (51), Obayemi et al. (52), Page et al. (53), Amimo et al. (54), Habinshuti et al. (55), Rawatet al. (56), Adewumi et al. (57), Pierzet al. (58), Lidofsky et al. (59), Dionet al. (60), Stewartet al. (61), Oscar Taperaet al. (62), Burrowes et al.(63), Maree et al. (64), Derejeet al. (65), Page et al. (66), Tapera et al. (67), Chapola et al. (68), Isaacson et al. (69), Kambhampati et al. (70)
Community-level barriers	Cultural acceptance and social norms; social support networks	Burrowes et al.(63), Tapera et al. (67), Kambhampati et al. (70), Atnafu et al. (71), Mensah et al. (72), Tapera et al. (73)
Health-system level barriers	Resource limitations; service delivery and quality; policy and governance	Randall et al. (50), Ngalla et al. (51), Lidofsky et al. (59), Burrowes et al. (63), Dereje et al. (65), Page et al. (66), Kambhampati et al. (70, Mensah et al. (72), Islam et al. (74, Fennie e t al. (75, Beltránbeltr et al. (76), Tapera et al. (77), Grover et al. (78, Shimels et al. (79), Tchounzou et al. (80), McCree et al. (81), de Fouw et al. (820)

Opportunities were also identified and thematically categorized to capture approaches for improving access to cervical cancer care and strengthening service delivery within existing healthcare systems. These themes emerged as facilitators and enablers of context-specific healthcare interventions, particularly those emphasizing multi-stakeholder engagement, community-driven approaches, and alternative service delivery strategies. Through an iterative thematic clustering process, these opportunities were grouped into two broader domains: methodological, which reflects approaches to service design and delivery, and technological, which highlights the use of innovative tools and technologies to enhance access and care provision. Because this review focuses on opportunities related to service delivery and access rather than on health policy or management systems, policy and management are not structured as separate analytical domains. Nevertheless, relatively few included studies identified policy and governance measures as enabling mechanisms to overcome barriers and improve cervical cancer care outcomes, alongside methodological and technological opportunities. The distribution and thematic focus of these opportunities are summarized in Table 4.

**Table 4:** Theme categorization for identified opportunities.

Themes for Opportunities	Sub-themes	Studies
Methodological	Community-driven health education, culturally informed health intervention, and multi-stakeholder engagement	Randall and Ghebre (50), Obayemi (51), Lidofsky et al. (59), Dion et al. (60), Chapola et al. (68), Islam et al. (73), Hussein et al (83), Adamu et al., (84)
Technological	Technology-enhanced health education, digital systems for patient navigation, and task decentralization	Ngalla et al. (51), Islam et al. (74), Beltránbeltr´et al. (76), Tapera et al. (77), Grover et al. (78), Addo-Lartey et al. (85), Adepoju et al. (86), Maimela et al.(87)

### 3.5 Identified barriers in access to cervical cancer care services

#### 3.5.1 Individual-level barriers

This theme captures how intersecting factors influence individuals’ ability and willingness to seek care services, with a particular focus on cervical cancer. These include demographic and socio-economic factors, such as age, educational attainment, economic status, and place of residence (49–55), as well as the acceptability and preferences for available services (56–58). Additionally, contextual barriers, including language barriers (49, 59, 60), place of residence (61), and cultural beliefs also prevent individuals from accessing available care services (59, 62).

A recurring theme across the literature was limited awareness and understanding of cervical cancer, including its risk factors and early symptoms (50, 63–65), and of available prevention and treatment options (51, 53, 66). Women and community members frequently demonstrated low health literacy, which contributed to delayed care-seeking, with many presenting only when symptoms had become severe. In several settings, low awareness intersected with fatalistic beliefs about cervical cancer. For instance, studies from Ethiopia (63) and Zimbabwe (67) reported a widespread belief that cervical cancer is incurable or the result of evil fate. These beliefs shaped initial responses to illness, with many women seeking help from religious leaders or traditional healers before engaging with biomedical services. Such pathways often resulted in significant delays in diagnosis and treatment, and reinforced poor outcomes.

Moreover, studies (68, 69) found that gender norms and partner dynamics were significant barriers to women’s access to screening and treatment services. These findings indicate that male partners actively discourage women from undergoing cervical cancer screening and treatment, particularly because the procedure requires undressing, which raises concerns about modesty and jealousy. Similarly, in the same study, a woman recounted her husband’s lack of support during her treatment, saying, ‘*They told me to wait for six weeks without having sex after treatment. When I told my husband, he asked: Is it possible for people who are married to stay for six weeks without sex? How is that even possible? After two weeks, I realized [chuckles] he was not going to manage*.’ This illustrates how women’s health decisions were embedded within unequal power relations, in which male partners’ expectations and lack of support constrained women’s ability to follow medical advice and remain engaged in care.

Evidence from Botswana and Zimbabwe indicated that financial constraints further compounded delays in seeking and receiving care (70). These constraints, particularly for women, created substantial difficulties in affording treatment, transportation, accommodation, and food when required to travel to urban centers for care (53, 68). A study (70), also reported that 70% of women (14 out of 20) preferred traditional healers to biomedical healthcare providers due to the high cost of formal healthcare services. This preference reflects not only economic hardship but also limited accessibility and affordability of cervical cancer care within public health systems, particularly for women living in rural or underserved areas.

#### 3.5.2 Community-level barriers

This theme captures how collectively held beliefs, social norms, and sources of authority within communities shaped attitudes toward cervical cancer and influenced decisions about prevention and treatment. Across studies, community-level meanings of cervical cancer extended beyond individual knowledge deficits, embedding the disease in moral, spiritual, and social interpretations that often discouraged engagement with biomedical services (71, 72).

Among these barriers, cultural beliefs and misinformation are particularly influential, as they can profoundly shape health behaviors and decisions about preventive and curative services. For example, a study from Ethiopia (63) described a woman who avoided screening because she believed her illness was caused by a curse and viewed cervical cancer as a death sentence. Such interpretations framed the disease as inevitable or spiritually determined, reducing the perceived value of preventive or early diagnostic services. Cultural beliefs and stigma linked to sexual morality further compounded these barriers. In Ghana, findings indicated that many men believed cervical cancer resulted from ‘too much sexual activity,’ reinforcing blame and social judgment toward affected women (70). These beliefs contributed to discrimination and social isolation, creating additional deterrents to seeking timely care.

Community mistrust of biomedical treatment was also evident, particularly regarding radiotherapy. Findings from a study in Zimbabwe (73) revealed widespread fear and resistance to cancer treatment, as illustrated by one account: *“In our community, people are very hesitant about radiotherapy. Many believe it involves placing heavy metals on the body that damage the veins, so they think it’s better to die at home than undergo treatment.”* This perception underscores how misconceptions and fear normalize avoidance of formal healthcare, even when services are available. Moreover, studies (63, 67) emphasized the influential role of community leaders and religious figures, who were often regarded as trusted authorities. Their endorsement of traditional or spiritual healing practices sometimes discouraged the use of biomedical care, making medical treatments socially unacceptable within certain community contexts.

#### 3.5.3 Health-system level barriers

This theme reflects how structural weaknesses in health systems constrained the delivery and continuity of cervical cancer prevention, diagnosis, and treatment services. Across studies, participants’ experiences revealed health systems marked by chronic resource shortages, fragmented care pathways, and uneven service distribution, which collectively undermined timely diagnosis, treatment completion, and patient trust (59). Insufficient public health education (59, 74), inconsistent availability of essential services such as vaccinations and screening programs (59, 72, 75), shortages of medicines and medical equipment (70, 76), limited access to trained and specialized personnel, fragmented care pathways, inadequate access to subsidized health insurance, and gaps in evidence-based policy implementation (66, 77) were widely reported barriers. These systemic limitations contributed to delayed detection and disrupted treatment trajectories, often resulting in women presenting with advanced-stage disease and experiencing interruptions in care (63, 65).

A shortage of the pathology workforce emerged as a critical structural barrier to timely cervical cancer diagnosis and treatment. A study by Randall et al. (50) highlighted severe shortages of pathologists, resulting in delays in histopathological confirmation and, in some cases, initiation of treatment without definitive pathological diagnosis. This uncertainty contributed to delays in treatment initiation and undermined patients’ confidence in recommended therapies. These findings are consistent with broader evidence from SSA, where the pathology workforce remains critically limited. The region has, on average, approximately 1 pathologist per 1,000,000 population (equivalent to 0.1 per 100,000 population), compared with approximately 5–12 pathologists per 100,000 population in many high-income countries. Such shortages substantially constrain timely diagnostic confirmation, treatment planning and initiation, and access to quality cancer care. Limited availability of gynecologic oncologists further constrained care options, increasing reliance on simple hysterectomy and leading patients to be more often treated surgically by a general gynecologist rather than with a radical hysterectomy, which would ideally be preferred but is very hard due to the very limited availability and accessibility of radiotherapy services. Furthermore, geographic inequalities in service availability compounded these barriers. Oncology centers, radiation therapy facilities, and trained healthcare workers were often concentrated in capital or major urban areas, requiring women from rural areas to travel long distances to access care (70, 76, 78).

Many women present with late-stage cancer, interrupt treatment when machines fail, and often cannot afford treatment and follow-up care, resulting in low survival rates. In line with this, a study conducted in Ethiopia (79) found that service costs were affordable in only 11.5% of cases. Although the country has developed a mixed payment model with contributions from the government, the private sector, and NGOs, 70% of patients may still need to pay out of pocket. Moreover, a study (76) reported that 10 of 54 African countries lacked access to radiation therapy machines, including Malawi, Chad, Cameroon, Guinea, Burundi, Cape Verde, the Democratic Republic of Congo, Seychelles, Eritrea, and Niger. A single radiation therapy center is common in these countries, as noted in Zimbabwe, Zambia, Uganda, and Ethiopia. Although radiation machines are available in urban, semi-urban, and capital cities, this creates uneven distribution and social exclusion in access to essential care services. This exacerbates care delays, underscores the consequences of centralized cancer care services, and the need for decentralized systems (80).

Individual case narratives illustrated how these system-level failures translated into adverse outcomes. One case reported by Ngalla et al. (51) involved a 37-year-old woman who faced significant delays in surgical care due to a shortage of gynecologic oncologists. Although she eventually underwent a hysterectomy, the specimen was not sent for pathology, and she later returned with complications after missing follow-up because of financial and travel barriers. By the time she returned the following year, she presented with advanced symptoms, including a large pelvic mass identified on CT scan. Ultimately, she was referred to palliative chemotherapy and continues to respond to treatment. This case highlights the critical impact of workforce shortages, delayed pathology services, and affordability issues on the timely diagnosis and effective management of cervical cancer in SSA. Meanwhile, a study by Grover et al. (78) underscores how both individual and system-level barriers contribute to significant delays in cervical cancer screening and diagnosis among women in Botswana. While individual fears and perceptions play a role, the most prominent delays stemmed from structural deficiencies, including shortages of testing equipment, inconsistent service availability, and inefficient communication of screening results. These system failures were particularly consequential for women, who are at higher risk and thus require consistent and timely screening (81). The repeated experience of not receiving results, even after multiple screenings, led some women to disengage from the screening process altogether. Similarly, a study by Tchounzou et al. (80) reported that among 110 cervical cancer patients, only 44 (fewer than half) were able to complete their planned treatment program, with discontinuation attributed to financial hardship, travel distance, and reliance on alternative care pathways. Notably, this scenario is also supported by de Fouw et al. (82) from the context of Uganda, of which 583 patients were included in the study, 86% presented at an advanced stage, and only 30% interrupted treatment and did not attend their planned appointments.

Together, these findings show that barriers to accessing and delivery of cervical care services are not the result of isolated individual choices but are produced through the interplay of community-level meanings, gendered social relations, and fragmented healthcare-system structures. Limited awareness and fatalistic beliefs, reinforced by stigma and community authority figures, intersect with structural constraints, including workforce shortages, centralized services, and financial hardship, to shape delayed care-seeking, treatment interruptions, and disengagement from services.

### 3.6 Identified opportunities

This theme captures facilitators and enablers identified, suggested, and recommended across studies that may help individuals engage with cervical cancer care services and overcome previously identified barriers. These opportunities are not presented as isolated interventions; rather, they are understood as relational approaches that align with local contexts, address mistrust of the healthcare system, and promote continuity of care.

#### 3.6.1 Methodological opportunities

Methodological opportunities emerged across studies as socially grounded strategies that facilitate engagement with cervical cancer care services by reshaping meanings, relationships, and decision-making processes. Rather than relying on a single solution, these approaches build trust, foster cultural alignment, and involve communities in the design and delivery of interventions. Community-driven health education was consistently identified as a key opportunity, particularly when delivered by trusted local actors, including religious and community leaders, health extension workers, medical professionals, and NGOs (60, 74, 83). These actors were perceived as credible messengers because of their embeddedness in local social structures and familiarity with cultural beliefs, norms, and languages. Their involvement enabled health messages about cervical cancer risk factors, symptoms, and screening to be communicated in ways that were locally meaningful and socially acceptable.

Such approaches were reported to reduce stigma and challenge fatalistic and moralized narratives surrounding cervical cancer by addressing misconceptions and reframing the disease as preventable and treatable when detected early (59, 84). Importantly, the effectiveness of these strategies is attributed not solely to the information provided but also to the relational processes through which knowledge was shared, fostering trust, encouraging dialogue, and legitimizing biomedical care within community contexts. Multi-stakeholder engagement further emerged as an enabling condition for sustained care engagement and continuity. Studies highlighted the value of including cervical cancer patients, healthcare providers, family members and caregivers, male partners, community workers, NGOs, and policymakers throughout the design and implementation of interventions (50, 52, 68). These inclusive, participatory approaches supported shared decision-making across the care continuum and could help align services with lived realities, particularly in contexts where health decisions were shaped by family dynamics and gendered power relations. Together, these methodological opportunities illustrate how interventions grounded in social relationships, cultural understanding, and participatory design could counteract stigma, rebuild trust, and support earlier and more sustained engagement with cervical cancer care.

#### 3.6.2 Technological opportunities

Digital technologies emerged across studies as an opportunity to address barriers related to distance, fragmented care pathways, and loss to follow-up in cervical cancer services. Unlike methodological opportunities, which operated primarily through embedding social relationships and cultural alignment, digital approaches improved coordination, communication, and continuity of care across the health system.

Digitally mediated health education was also identified as an important opportunity for improving access and service delivery. Localized health literacy initiatives delivered through radio broadcasts, mobile messaging, and other digital platforms enabled cervical cancer information to be communicated in culturally relevant languages and formats, extending outreach beyond health facilities and overcoming language and literacy barriers (85). Such approaches were perceived as particularly valuable for reaching women in rural or underserved communities. Reminder systems and call-based follow-up mechanisms further emerged as enabling practices by supporting appointment attendance and reducing missed follow-up visits (74). In settings where women frequently experienced delays in receiving test results or discontinuity between screening and treatment, digital patient navigation and tracking systems helped maintain engagement and support timely progression along the care continuum (51, 76–78).

Technology-enabled decentralization of diagnostic services was also highlighted as a potential opportunity for equitable access. Studies described how digital solutions could support task-sharing, remote interpretation of diagnostic results, and coordinated data sharing between peripheral and central facilities, thereby reducing diagnostic delays and mitigating the effects of centralized service models (86, 87). Together, these findings indicate that digital technologies were understood to enable cervical cancer care by strengthening system connectivity and continuity, complementing rather than replacing relational and community-based approaches.

## 4 Discussion

### 4.1 Interconnected barriers across levels

This review highlights an interconnected set of barriers that affect individuals’ and communities’ access to cervical cancer care and the performance of health systems in delivering these services. These barriers include limited awareness and knowledge of cervical cancer, negative attitudes, socio-economic challenges, and geographic disparities at the individual and community levels, alongside health system limitations such as inadequate infrastructure, workforce shortages, and ineffective implementation of policies and guidelines. The findings indicate that barriers to cervical cancer care emerge not as isolated factors but as a cascading system of influence, in which upstream health-system constraints shape community perceptions and norms, which are then internalized at the individual level and reflected in health-seeking behaviors.

At the health-system level, persistent shortages and uneven distribution of HPV vaccines and screening services disproportionately favor urban communities. Research shows that HPV vaccine rollout in SSA faces substantial supply-side challenges, including high procurement costs, weak funding, and inadequate cold chain infrastructure, which limit overall vaccine availability and coverage in national immunization programs (88). When vaccines and screening services are available, they are concentrated in urban centers, leaving rural women and girls with scarce or no access to primary prevention. This rural–urban divide in service availability cascades into awareness and health education. Because many public health campaigns and educational programs are also urban-based and limited in scope, women and communities in rural areas remain less informed about cervical cancer, risk factors, and preventive and curative measures. These systemic weaknesses often lead individuals and communities to seek alternative care options, such as traditional healers, spiritual practices, or private healthcare providers (77). Consistent with prior research, limited health education is associated with lower health literacy, reduced awareness, and inadequate understanding of disease risk factors and preventive and curative measures (89). These findings show that limited health literacy and a lack of community-level education contribute to misconceptions about cervical cancer, stigmatization of services, and low perceived need for preventive care.

Furthermore, geographic distance and socioeconomic barriers amplify this cycle, as long distances to facilities and transportation costs further diminish uptake of available services (90–92). Access in this context is constrained not only by the availability of services but also by residential location, limited health literacy, and socio-economic barriers that discourage women and the community from seeking care. These systemic failures not only limit service availability but also undermine confidence in the healthcare system. Consequently, the cervical cancer care continuum remains fragmented and unclear, compounded by bureaucratic and inefficient referral systems, a phenomenon commonly reported in LRS, where weak coordination between levels of care undermines continuity and timeliness of services (93). These systemic shortcomings have ripple effects at the community level, where shared experiences of delays, unclear guidance, and inconsistent care foster mistrust in formal healthcare services. In such contexts, weakened trust in health systems can reinforce culturally grounded narratives about disease causation and treatment, especially when services fail to provide clear information and communication, timely diagnosis, and positive care experiences.

**Figure 5:**
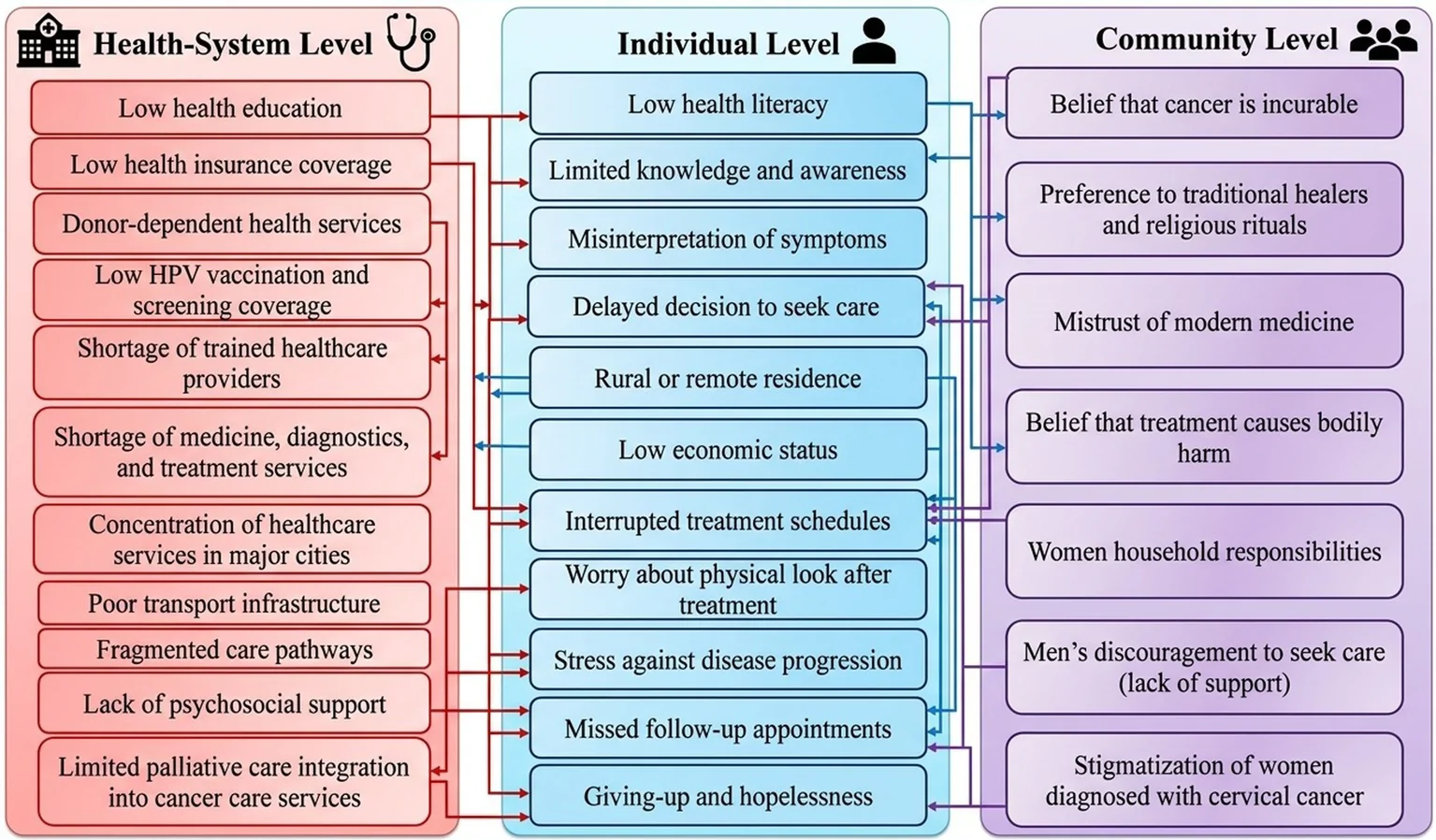
Interconnected barriers shaping cervical cancer care service delivery and access in SSA.

At the individual level, these health-system and community-level shortcomings manifest as low health literacy, entrenched misconceptions, and delayed care-seeking behaviors. Limited awareness of cervical cancer symptoms, prevention strategies, and treatment options is not merely an individual informational deficit but reflects broader structural constraints, including weak prevention programs, fragmented service delivery, and limited access to reliable health education. For example, as of 2019, only 20% of countries in SSA, including Botswana, Cameroon, Ethiopia, Kenya, Malawi, South Africa, Rwanda, and Zimbabwe, had established national cervical cancer screening programs, and in most of these settings, population-level screening coverage remained below 10% (94). Although this finding reflects the situation during the study period, several countries have since strengthened their cervical cancer prevention policies. For instance, Nigeria has adopted the WHO Global Strategy to Accelerate the Elimination of Cervical Cancer and launched a nationwide cervical cancer screening program as part of its 2026–2030 National Strategic Plan, demonstrating ongoing regional efforts to expand screening and improve early detection (95). Nevertheless, implementation challenges remain, and equitable access to screening services continues to vary substantially across SSA. In such contexts, individuals have fewer opportunities to encounter accurate information, preventive services, or positive care experiences. Consistent with this interpretation, previous studies show that cervical cancer screening and treatment uptake are strongly influenced by educational attainment, age, and distance to health facilities, highlighting how structural and geographic barriers translate into individual health-seeking behaviors (25, 96). Collectively, these findings demonstrate that cervical cancer prevention and control efforts in SSA remain severely constrained by the interplay of healthcare-system limitations, socioeconomic inequities, and individual decision-making processes. Figure 5 below illustrates how these factors intersect and reinforce one another, emphasizing the need for integrated, multilevel interventions to address them effectively.

### 4.2 Opportunities for (re)designing of healthcare interventions

**Figure 6:**
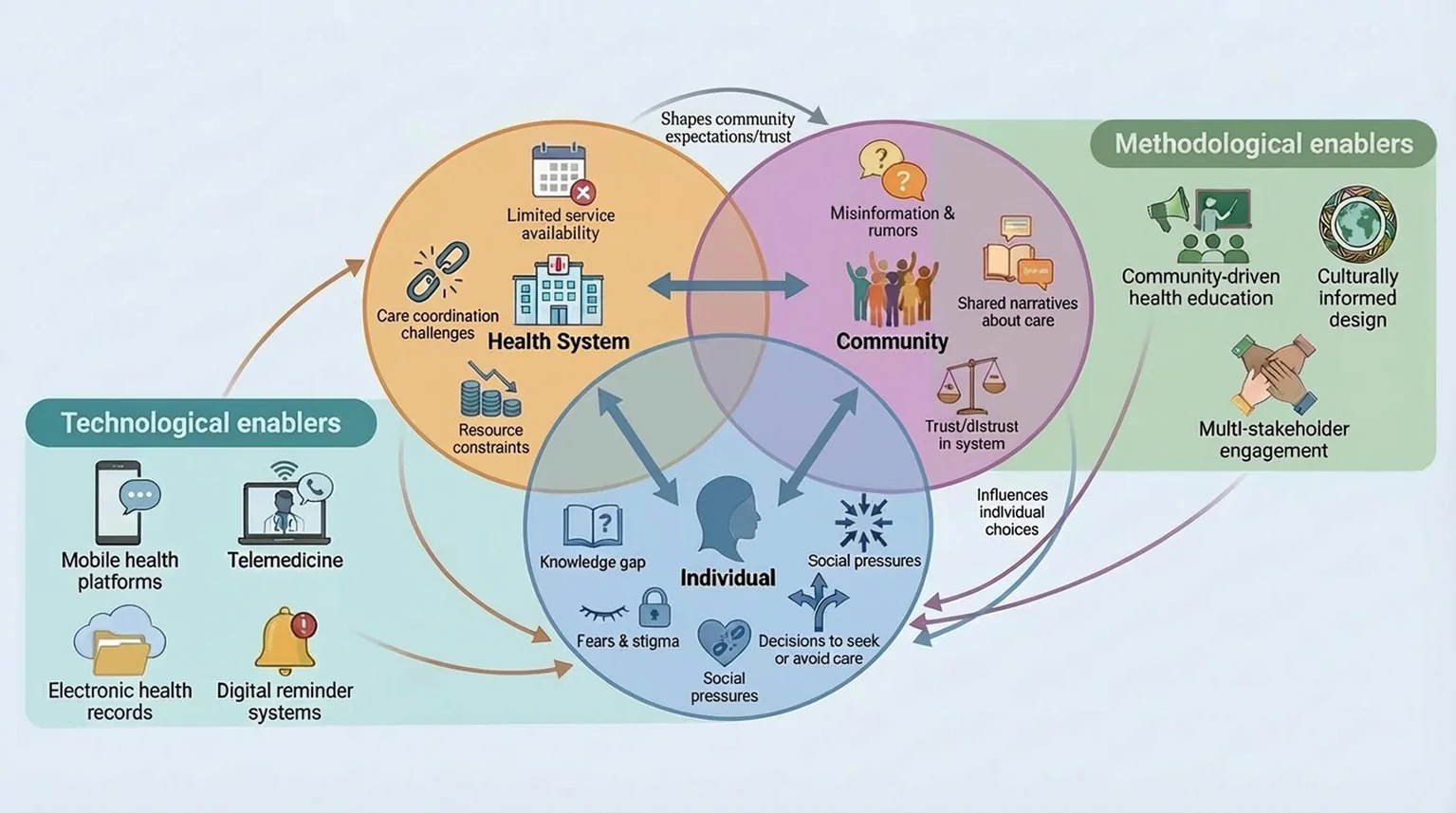
Interrelationships among multilevel barriers and opportunities shaping cervical cancer care access and service delivery in SSA.

Despite the substantial barriers identified, this review highlights several opportunities for improvements in service delivery and access to cervical cancer care. In particular, methodological approaches and digital technologies emerge as cross-cutting strategies with demonstrated potential to strengthen healthcare service delivery and access. Community-driven health education, culturally informed intervention design, and multi-stakeholder engagement throughout intervention design have been widely shown to address gaps in awareness, trust, and service uptake in LRS (97–99). For instance, engaging community and religious leaders, healthcare providers, researchers, and policymakers would be effective, as these trusted figures could help to overcome cultural barriers, improve outreach, counter misinformation, reduce stigma, build trust, and tailor culturally sensitive messages (100). Ultimately, these approaches could improve health literacy and foster shared decision-making across the continuum of care. Complementing these approaches, digital technologies continue to emerge as a powerful opportunity for care coordination, communication, patient navigation, and follow-up. Evidence from both cancer and non-cancer contexts suggests that technology-enabled decentralization of care services could mitigate workforce shortage, distance, and economic challenges in expanding access to care (101–105). Digital health platforms such as telemedicine (101, 106), electronic health records (107), and digital reminder systems (108, 109) have been shown to improve health education and appointment adherence, facilitate timely referrals, reduce diagnostic delays, and enhance continuity of care across a range of health conditions. When these tools are designed with culturally appropriate language and locally relevant delivery channels, they could effectively bridge geographic, logistical, and informational barriers, thereby reinforcing the acceptability and effectiveness of health interventions (see Figure 6).

Taken together, these findings, as shown in Figure 6 above, illustrate the dynamic and deeply interconnected nature of barriers to cervical cancer care across health-system, community, and individual levels. Health-system constraints such as limited service availability, resource limitations, and the concentration of services in urban centers not only restrict access but also shape community expectations, trust, and shared narratives about care. These community-level interpretations, in turn, influence how women perceive risk, evaluate available options, and ultimately decide whether to seek care. At the individual level, awareness and knowledge gaps, fears, and social pressures emerge as both consequences and drivers of these broader structural and social dynamics, underscoring that access to care is not solely determined by physical availability but is embedded within a complex, lived reality in which systems, communities, and individuals continuously interact.

Addressing these multilayered barriers requires integrated, contextually grounded approaches. Methodological opportunities such as community-driven health education, culturally informed healthcare intervention design, and multi-stakeholder engagement could strengthen trust, reduce misinformation and stigma, and improve health literacy by aligning interventions with local beliefs and experiences. For example, engaging community and religious leaders in co-designing initiatives could enhance the acceptability and uptake of available services. Complementing these efforts, technological opportunities, including mobile health platforms, telemedicine, electronic health records, and digital reminder systems, could help address structural barriers by decentralizing care beyond urban centers, extending services to remote and underserved populations, improving care coordination, reducing diagnostic delays, and supporting continuity of care. Together, these strategies highlight the importance of multilevel interventions that bridge structural, social, and individual barriers, ultimately advancing a more equitable and effective cervical cancer care system.

An important finding of this review is the limited emphasis on policy and governance as enabling mechanisms for improving access to cervical cancer care. While the literature frequently highlighted technological innovations and methodological approaches to strengthen service delivery and improve access, comparatively few studies (3 of 38) examined the policy and governance frameworks needed to support their adoption, implementation, and scale-up alongside these opportunities (52, 62, 71). This gap is important because technological and methodological innovations alone are unlikely to achieve sustained impact without supportive policies, sustainable financing, and accountability mechanisms. Policy and governance structures play a central role in integrating evidence-based interventions into routine health systems, coordinating implementation across levels of care, and promoting equitable access to cervical cancer services. The limited policy-focused evidence identified in this review, therefore, represents an important research gap that may constrain efforts to translate promising innovations into sustainable, large-scale cervical cancer control programs across SSA.

### 4.3 Evidence gap and implications

This narrative review reveals substantial gaps in research on the accessibility of cervical cancer care across the continuum of care. Most studies (27 of 38) focus on screening, diagnosis, and treatment, leaving knowledge and awareness, as well as post-treatment care, underexamined. Furthermore, research predominantly identifies barriers to access without exploring how these barriers could inform the design of more effective healthcare services and interventions. The absence of a design-oriented perspective further limits the translation of evidence into innovative, contextually appropriate interventions.

Addressing these gaps has critical implications. By reframing barriers not merely as obstacles but as actionable inputs for human-centered and digitally enabled solutions, interventions could be tailored to the realities of service users and providers in SSA. For instance, while digital reminders or telemedicine platforms mitigate missed appointments and geographic barriers, their success depends on health literacy, language, trust, gendered barriers to device access, and integration with the existing care continuum. This underscores the importance of engaging multiple stakeholders, including patients, caregivers, providers, and community leaders, in participatory and co-design processes to ensure acceptability, usability, and sustainability. Overall, bridging these research gaps through a design-oriented approach could inform healthcare interventions that enhance access across all phases of cervical cancer care rather than focusing solely on single clinical outcomes.

### 4.4 Limitations of the study

This review is limited by the timing of the literature search, which was conducted in May 2025 and was not updated before manuscript submission in May 2026. As a result, relevant studies published during this interval were not captured. Although this review synthesizes the evidence available at the time of the search, the omission of more recent publications may have excluded new findings, policy developments, or implementation experiences that could further inform understanding of barriers and opportunities for improving access to cervical cancer care in SSA. Future updates of this review should incorporate recently published evidence to maintain the currency of the findings and recommendations.

## 5 Conclusion

This narrative review demonstrates that access to cervical cancer care in SSA is shaped by interacting sociocultural, individual, and healthcare system structural barriers that extend beyond supply and service availability. These multilevel barriers contribute to low health literacy, delayed diagnosis, poor outcomes, and fragmented continuity of care. Important research gaps remain in primary prevention, follow-up, and palliative care, and relatively few studies treat these barriers as opportunities to inform contextually grounded, responsive healthcare interventions. These findings underscore the need for integrated, multilevel strategies that strengthen coordination across the care continuum. Context-specific, human-centered, and co-designed interventions that actively engage local communities, incorporate technological innovations, and promote multidisciplinary collaboration may play an important role in improving access, service delivery, continuity of care, and cervical cancer outcomes in SSA. This review makes two main contributions to research and design on cervical cancer care interventions: first, it provides an evidence-based mapping of barriers influencing access and service delivery across the cervical cancer care continuum, and second, it advances a design-oriented perspective by reframing these barriers as actionable inputs and translating them into implications for human-centered, technology-enabled healthcare interventions.

## Footnotes

1
https://app.covidence.org

## Supplementary material

Supplementary Table 1 Data extraction process and characteristics of included studies.
