# Supplementary material for: Access to cervical cancer care in sub-Saharan Africa: a narrative review of barriers and opportunities
Source: Front Public Health. 2026 Aug 18;14:1881652. doi: 10.3389/fpubh.2026.1881652 (PMC13529692; doi:10.3389/fpubh.2026.1881652)
Supplement: Supplementary file 1 [file Table_1.DOCX]

| Title and year | Country | Study design | Aim | Population | Care-continuum stage | Key findings: barriers and opportunities |
| --- | --- | --- | --- | --- | --- | --- |
| 1. The effect of health education on the knowledge, attitude, and uptake of free Pap-smear among female teachers in Birnin-Kebbi, North-Western Nigeria. (2012) | Nigeria | Quasi-experimental educational intervention | Assess whether health education improves knowledge, attitudes, and uptake of free Pap-smear screening. | Female teachers in Birnin-Kebbi, north-western Nigeria. | Prevention and screening | Barriers: limited baseline knowledge and low screening uptake. Opportunities: targeted education combined with free screening increased awareness and participation. |
| 2. Effectiveness of a culturally tailored text messaging program for promoting cervical cancer screening in Accra, Ghana: A quasi-experimental trial. (2024) | Ghana | Quasi-experimental trial | Evaluate a culturally tailored text-messaging program to promote cervical cancer screening. | Screening-eligible women in Accra. | Prevention and screening | Barriers: limited awareness, competing priorities, and difficulty sustaining screening attendance.  Opportunities: culturally tailored SMS education and reminders can improve screening engagement. |
| 3. Targeting women with free cervical cancer screening: challenges and lessons learnt from Osun state, southwest Nigeria. (2016) | Nigeria | Program evaluation / cross-sectional assessment | Describe challenges and lessons from offering free cervical cancer screening in Osun State. | Women attending a free screening program in south-west Nigeria. | Screening | Barriers: low awareness, limited service capacity, and weak follow-up after screening.  Opportunities: free outreach screening, community mobilization, and stronger referral systems. |
| 4.  Women’s experiences and acceptability of self-administered, home-delivered, intravaginal 5-Fluorouracil cream for cervical precancer treatment in Kenya. (2025) | Kenya | Qualitative study | Explore women's experiences and acceptability of home-delivered, self-administered intravaginal 5-fluorouracil for cervical precancer. | Women receiving treatment for cervical precancer in Kenya. | Treatment and follow-up | Barriers: concerns about side effects, correct self-administration, privacy, and follow-up.  Opportunities: home-based treatment may reduce travel and improve convenience when supported by counseling and monitoring. |
| 5. Impact of per capita income on the effectiveness of school-based health education programs to promote cervical cancer screening uptake in southern Mozambique. (2018) | Mozambique | School-based health-education intervention / quantitative study | Assess whether income influences the effectiveness of school-based education in promoting cervical cancer screening. | Women reached through a school-linked health-education program in southern Mozambique. | Prevention and screening | Barriers: poverty and limited access reduced screening uptake. Opportunities: school-based education and affordable or free screening can increase participation, especially when adapted to socioeconomic circumstances. |
| 6. Drivers of cervical cancer prevention and management in sub-Saharan Africa: a qualitative synthesis of mixed studies. (2024) | Multi-country SSA | Qualitative evidence synthesis | Synthesize drivers and barriers influencing cervical cancer prevention and management across sub-Saharan Africa. | Women, health workers, managers, and other stakeholders were represented in 60 studies across 16 SSA countries. | Entire care continuum | Barriers: low awareness, stigma, costs, distance, infrastructure gaps, and weak policies.  Opportunities: education, provider recommendations, female providers, community models, and integration with HIV and reproductive health services. |
| 7. Access to radiation therapy and related clinical outcomes in patients with cervical and breast cancer across Sub-Saharan Africa: A systematic review.  (2023) | Multi-country SSA | Systematic review | Assess access to radiotherapy and related clinical outcomes for cervical and breast cancer across SSA. | Evidence from 96 articles on patients requiring radiotherapy across several SSA countries. | Treatment and palliative care | Barriers: severe radiotherapy and workforce shortages, long travel times, high out-of-pocket costs, and equipment failures.  Opportunities: expand radiotherapy capacity, financing, workforce training, maintenance, and regional referral networks. |
| 8. Perceptions of cervical cancer care among Ethiopian women and their providers: a qualitative study (2022) | Ethiopia | Qualitative study | Explore perceptions of cervical cancer care among Ethiopian women and healthcare providers. | Women with cervical cancer and healthcare providers in Ethiopia. | Diagnosis and treatment | Barriers: delayed presentation, fragmented referrals, poor communication, stigma, and financial constraints. Opportunities: patient navigation, clearer communication, decentralized services, and provider training. |
| 9. Barriers to follow-up after an abnormal  cervical cancer screening result and the role of male partners: A qualitative study. (2021) | Malawi | Qualitative study | Explore barriers to follow-up after abnormal screening and the role of male partners. | Women with abnormal cervical screening results and relevant partners/providers in Malawi. | Screening follow-up and treatment linkage | Barriers: transport costs, fear, limited partner support, poor communication, and service delays.  Opportunities: male partner engagement, counseling, reminder systems, and coordinated referrals. |
| 10. Screening for cervical cancer among women in five countries in sub-Saharan Africa: Analysis of the role played by distance to health facility and socio-demographic factors. (2023) | Multi-country SSA | Cross-sectional secondary analysis | Examine how distance to health facilities and sociodemographic factors affect cervical cancer screening in five SSA countries. | Women of screening age are represented in the national household survey datasets from five SSA countries. | Screening | Barriers: long distance, rural residence, lower education, and socioeconomic disadvantage.  Opportunities: decentralized and mobile screening, outreach, and targeted support for underserved women. |
| 11. Evaluation of video-assisted HPV education in government-supported clinics in Western Kenya. (2023) | Kenya | Pre-post educational evaluation | Evaluate video-assisted HPV education in government-supported clinics in western Kenya. | Women attending public clinics in western Kenya. | Prevention and screening | Barriers: limited knowledge of HPV and misconceptions. Opportunities: brief video-assisted education delivered in clinics improved knowledge and may support informed screening decisions. |
| 12. Patient perspectives on delays in cervical cancer screening and follow-up care in Botswana: a mixed methods study (2022) | Botswana | Mixed-methods study | Identify patient-reported factors contributing to delays in screening, diagnosis, and follow-up. | Women receiving cervical cancer care in Botswana. | Screening, diagnosis, and follow-up | Barriers: low HPV knowledge, stigma, transport burden, delayed results, weak communication, and scarce radiotherapy/testing capacity. Opportunities: awareness campaigns, navigation, SMS reminders, social support, and better tracking. |
| 13. Factors associated with loss to follow-up among cervical cancer patients in Rwanda (2020) | Rwanda | Retrospective cohort study | Identify factors associated with loss to follow-up among patients with cervical cancer. | Women diagnosed with cervical cancer and receiving care in Rwanda. | Treatment and follow-up | Barriers: advanced disease, travel distance, financial hardship, and weak follow-up systems.  Opportunities: patient tracking, navigation, transport support, and decentralized follow-up. |
| 14. Assessing the influence of the health system on access to cervical cancer prevention, screening, and treatment services at public health centers in Addis Ababa, Ethiopia. (2024) | Ethiopia | Cross-sectional health-system assessment | Assess how the health system influences access to prevention, screening, and treatment services in Addis Ababa. | Healthcare facilities, health workers, and service users in public health centers in Addis Ababa. | Prevention, screening, diagnosis, and treatment | Barriers: inadequately trained staff, supplies, equipment, referral pathways, and service integration.  Opportunities: workforce development, reliable supply chains, integrated services, and strengthened referral systems. |
| 15. A Qualitative Exploration of Barriers to Treatment Among HPV-Positive Women in a Cervical Cancer Screening Study in Western Kenya. (2023) | Kenya | Qualitative study | Explore barriers to treatment among HPV-positive women enrolled in a cervical cancer screening study. | HPV-positive women referred for treatment in western Kenya. | Screening follow-up and precancer treatment | Barriers: cost, distance, fear, competing responsibilities, poor understanding, and service delays.  Opportunities: counseling, transport support, same-day services, reminders, and patient navigation. |
| 16. Health care provider perceptions of facilitators and barriers to human papillomavirus vaccination delivery in five countries. (2021) | Multi-country | Qualitative multi-country study | Explore healthcare providers' perceptions of barriers and facilitators to HPV vaccine delivery across five countries. | Healthcare providers are involved in HPV vaccination in five countries. | Primary prevention (HPV vaccination) | Barriers: supply and staffing constraints, misinformation, weak coordination, and difficulties reaching adolescents. Opportunities: school-based delivery, community engagement, provider training, and reliable vaccine supply. |
| 17. Barriers to early diagnosis and treatment of cervical cancer in Addis Ababa, Ethiopia: qualitative study (2025) | Ethiopia | Exploratory sequential mixed-methods study (survey and interviews) | Examine barriers and facilitators to cervical cancer screening from the perspectives of women and healthcare workers | 215 screening-eligible women and 17 healthcare workers from three government hospitals | Prevention and screening | **Barriers:** low awareness and knowledge of cervical cancer and screening; financial cost of screening; absence of a national screening program; limited availability of screening services and trained personnel; inadequate equipment/resources; transportation and accessibility challenges; fear, embarrassment, stigma, and cultural beliefs.  **Opportunities:** community education and awareness campaigns; integration of screening into routine healthcare services; provider training; policy support and sustainable national screening programs; improved health system resources and service organization |
| 18. A Study of Barriers to Cervical Cancer Care in Sub-Saharan Africa. (2023) | Multi-country SSA | Multi-institutional cross-sectional study | Characterize barriers to cervical cancer care across institutions in sub-Saharan Africa. | Patients and/or oncology providers from multiple cancer centres in SSA. | Diagnosis and treatment | Barriers: delayed referral, high costs, limited radiotherapy and diagnostics, staffing shortages, and long travel. Opportunities: regional coordination, expanded capacity, financial protection, navigation, and standardised pathways. |
| 19. Development and Implementation of a Culturally Appropriate Education Program to Increase Cervical Cancer Screening among Maasai Women in Rural Tanzania. (2019) | Tanzania | Community-based educational intervention | Develop and implement a culturally appropriate programme to increase screening among rural Maasai women. | Maasai women in rural Tanzania. | Prevention and screening | Barriers: low awareness, cultural concerns, distance, and limited screening availability.  Opportunities: culturally tailored education, community leadership involvement, and locally accessible screening. |
| 20. Service Accessibility as a Determinant of Cervical Cancer Screening in Gwanda District, Zimbabwe (2023) | Zimbabwe | Mixed-methods cross-sectional study | Assess how service accessibility influences cervical cancer screening uptake in Gwanda District. | 609 screening-eligible women, focus-group participants, and healthcare workers. | Screening | **Barriers**: distant facilities, inconsistent outreach, transport costs, rural disadvantage, and limited awareness.  **Opportunities**: regular outreach, decentralised screening, community education, and affordable referral. |
| 21. The impact of decentralizing colposcopy services from tertiary-level to primary-level care in inner-city Johannesburg, South Africa: A before and after study. (2019) | South Africa | Retrospective before-and-after study | Evaluate the effect of decentralising colposcopy from tertiary to primary care. | Women with abnormal Pap-smear results before and after decentralisation in Johannesburg. | Diagnosis and treatment of precancer | Barriers: centralised services, travel burden, and referral delays. Opportunities: primary-care colposcopy increased access while maintaining service quality. |
| 22. The psychosocial implications of cervical cancer in women living in sub-Saharan Africa (2025) | Multi-country SSA | Integrative or narrative review | Synthesize psychosocial implications of cervical cancer among women in SSA. | Published evidence concerning women living with cervical cancer in SSA. | Treatment, survivorship, and palliative care | Barriers: stigma, fear, depression, relationship strain, financial distress, and inadequate psychosocial support.  Opportunities: counselling, peer and family support, stigma reduction, and integration of psychosocial care. |
| 23. Expanding Cervical Cancer Screening and Treatment in Tanzania: Stakeholders’ Perceptions of Structural Influences on Scale-up. (2015) | Tanzania | Qualitative stakeholder study | Explore structural influences on scaling up cervical cancer screening and treatment. | Policy makers, programme managers, providers, and other stakeholders in Tanzania. | Screening and treatment | Barriers: limited funding, workforce and equipment shortages, weak referral and data systems, and competing priorities.  Opportunities: task shifting, integration, decentralisation, partnerships, and stronger planning. |
| 24. Challenges faced in managing cervical cancer patients who present post-operatively with more advanced disease in LMICs: Case studies from Cameroon. (2024) | Cameroon | Qualitative descriptive case series | Illustrate challenges in managing women who present post-operatively with more advanced cervical cancer. | Three women with cervical cancer treated in Cameroon. | Diagnosis, treatment, and palliative care | Barriers: incomplete staging, inappropriate early surgery, limited pathology/imaging, radiotherapy shortages, cost, and poor follow-up. Opportunities: insurance, diagnostic capacity, multidisciplinary review, telemedicine, and health education. |
| 25. Impact of per capita income on cervical cancer: improving equitable access to preventive services (2015) | Nigeria | Programme or policy report | Describe approaches to improve equitable access to cervical cancer preventive services in Nigeria. | Nigerian women eligible for prevention and screening services. | Prevention and screening | Barriers: inequitable access, low awareness, cost, and inadequate service coverage.  Opportunities: free or subsidised prevention, outreach, education, policy support, and stronger referral. |
| 26. Equity in access and utilization of cervical cancer treatment and palliation services in Harare, Zimbabwe (2019) | Zimbabwe | Mixed-methods study / report | Examine equity in access and use of cervical cancer treatment and palliation services in Harare. | Women with cervical cancer and health-system stakeholders in Harare. | Treatment and palliative care | Barriers: high out-of-pocket costs, urban concentration, delayed referrals, medicine/equipment shortages, and rural exclusion. Opportunities: subsidies, decentralisation, simplified referrals, workforce training, and patient tracking. |
| 27. Patient factors affecting successful linkage to treatment in a cervical cancer prevention program in Kenya: A prospective cohort study. (2019) | Kenya | Prospective cohort study | Identify patient factors associated with timely linkage to treatment after a positive hrHPV screen. | 505 hrHPV-positive women aged 25-65 years in rural western Kenya. | Screening follow-up and treatment linkage | Barriers: distance, cost, limited education, anxiety, and lack of encouragement.  Opportunities: counselling, transport support, reminders, navigation, and rapid treatment appointments. |
| 28. Systems-level barriers to treatment in a cervical cancer prevention program in Kenya: Several observational studies. (2020) | Kenya | Observational programme study | Identify system-level barriers to treatment in a cervical cancer prevention programme. | Women referred for treatment after positive screening in western Kenya, plus programme records/providers. | Screening follow-up and precancer treatment | Barriers: referral fragmentation, limited appointments, stock-outs, communication failures, and travel burden.  Opportunities: same-day treatment, integrated tracking, referral coordination, and reliable supplies. |
| 29.  Acceptability of self-sampling for cervical cancer screening among women living with HIV and HIV-negative women in Limbé, Cameroon. (2020) | Cameroon | Cross-sectional acceptability study | Assess acceptability of cervical self-sampling among women living with and without HIV. | Women living with HIV and HIV-negative women in Limbé, Cameroon. | Screening | Barriers: limited awareness, concerns about self-collection accuracy, discomfort, and follow-up access.  Opportunities: high acceptability, privacy, convenience, counselling, and community-based self-sampling. |
| 30. Challenges in prevention and care delivery for women with cervical cancer in sub-Saharan Africa. (2016) | Multi-country SSA | Narrative review | Review challenges in prevention and care delivery for women with cervical cancer in SSA. | Published evidence on women at risk of or living with cervical cancer across SSA. | Entire care continuum | Barriers: low vaccination and screening coverage, late diagnosis, limited treatment capacity, workforce shortages, and financial constraints.  Opportunities: vaccination, screen-and-treat, task shifting, integrated HIV services, and health-system investment. |
| 31. Acceptability and preferences for self‐collected screening for cervical cancer within health systems in rural Uganda: A mixed‐methods approach. (2021) | Uganda | Mixed-methods study | Assess acceptability and preferences for self-collected cervical cancer screening in rural health systems. | Women eligible for cervical screening and relevant health-system stakeholders in rural Uganda. | Screening | Barriers: distance, embarrassment, limited information, and concerns about test accuracy and follow-up.  Opportunities: self-collection was acceptable and could extend screening when paired with education and reliable referral. |
| 32. Access to advanced healthcare services and its associated factors among patients with cervical cancer in Addis Ababa, Ethiopia. (2024) | Ethiopia | Analytical cross-sectional study | Assess access to advanced healthcare services and factors associated with access among cervical cancer patients. | 391 women receiving treatment or follow-up at two referral hospitals in Addis Ababa. | Diagnosis, treatment, and follow-up | Barriers: centralised services, medicine stock-outs, unaffordable care, fragmented pathways, and limited radiotherapy/diagnostics. Opportunities: supply-chain improvement, insurance reform, navigation, decentralisation, and community education. |
| 33. Modeling spatial access to cervical cancer screening services in Ondo State, Nigeria. (2020) | Nigeria | Geospatial modelling study | Model geographic access to cervical cancer screening services in Ondo State. | Screening facilities and the female population of Ondo State represented in spatial datasets. | Screening | Barriers: uneven facility distribution, long travel times, and rural geographic inequity.  Opportunities: use spatial planning to locate new services, deploy mobile clinics, and prioritise underserved areas. |
| 34. Health system constraints affecting treatment and care among women with cervical cancer in Harare, Zimbabwe. (2019) | Zimbabwe | Mixed-methods health-system study | Explore how health-system organisation and capacity affect treatment and care for women with cervical cancer. | 134 patients, 78 health workers, focus groups, and 16 in-depth interviews in Harare. | Diagnosis and treatment | Barriers: high costs, urban concentration, slow referrals, staff and equipment shortages, and drug stock-outs.  Opportunities: rural expansion, subsidies, referral reform, training, mobile coordination, and patient tracking. |
| 35. Limited knowledge and access to palliative care among women with cervical cancer: An opportunity for integrating oncology and palliative care in Zimbabwe. (2020) | Zimbabwe | Cross-sectional mixed-methods study | Assess knowledge of and access to palliative care among women with cervical cancer. | Women with cervical cancer receiving care in Zimbabwe. | Palliative care | Barriers: limited knowledge, late referral, scarce services, pain-medicine shortages, and financial/geographic barriers.  Opportunities: integrate palliative care with oncology, educate patients/providers, and improve opioid and service availability. |
| 36. Determinants of access and utilization of cervical cancer treatment and palliative care services in Harare, Zimbabwe. (2019) | Zimbabwe | Mixed-methods analytical study | Identify determinants of access to and utilisation of cervical cancer treatment and palliative care. | Women with cervical cancer and relevant providers in Harare. | Treatment and palliative care | Barriers: poverty, distance, delayed referral, insufficient staffing/equipment, and inadequate insurance. Opportunities: financial protection, decentralized care, clear referral pathways, and integrated oncology-palliative services. |
| 37. Patients Lost to Follow-Up for Cervical Cancer in the Limbe Regional Hospital. (2019) | Cameroon | Retrospective cohort/record review | Describe the magnitude and determinants of loss to follow-up among cervical cancer patients at Limbé Regional Hospital. | Women diagnosed or treated for cervical cancer at Limbé Regional Hospital. | Treatment and follow-up | Barriers: weak tracking, financial and travel constraints, delayed care, and limited communication.  Opportunities: patient registries, active tracing, navigation, reminders, and decentralised follow-up. |
| 38. Perceptions of barriers and facilitators for cervical cancer screening from women and healthcare workers in Ghana: Applying the Dynamic Sustainability Framework (2025) | Ghana | Phenomenological qualitative study (in-depth interviews) | To explore patients' lived experiences and identify barriers to early diagnosis and treatment of cervical cancer. | 40 women with cervical cancer (24 with diagnostic delay and 16 with treatment delay) | Diagnosis and treatment | **Barriers:** inadequate knowledge of cervical cancer symptoms and available services; reliance on traditional healers and religious practices before seeking medical care; fear of treatment side effects and poor adherence; financial hardship; limited diagnostic and treatment infrastructure; shortage of trained healthcare providers; poor quality of care.  **Opportunities:** strengthen public awareness; expand screening, diagnostic and treatment infrastructure; improve provider training and quality of care; reduce financial barriers to diagnosis and treatment. |

Abbreviations: HPV, human papillomavirus; hrHPV, high-risk human papillomavirus; SSA, sub-Saharan Africa. Opportunities include facilitators, enabling factors, intervention effects, or recommendations reported in the included studies during the extraction of material.
